# Shiga toxin (Stx) type 2‐induced increase in O‐linked N‐acetyl glucosamine protein modification: a new therapeutic target?

**DOI:** 10.15252/emmm.202115389

**Published:** 2021-12-22

**Authors:** Rebecca A Bova, Angela Melton‐Celsa

**Affiliations:** ^1^ Department of Microbiology and Immunology Uniformed Services University of the Health Sciences Bethesda MD USA; ^2^ The Geneva Foundation Tacoma WA USA

**Keywords:** Digestive System, Microbiology, Virology & Host Pathogen Interaction, Post-translational Modifications & Proteolysis

## Abstract

Shiga toxin (Stx)‐producing *Escherichia coli* (STEC) causes bloody diarrhea, which may progress to the potentially fatal hemolytic uremic syndrome (HUS). Development of HUS after STEC infection is dependent on Stx, and is particularly linked to Stx type 2a, Stx2a (Melton‐Celsa, 2014; Scheutz, 2014). In this issue of *EMBO Molecular Medicine*, Lee *et al* report that O‐linked N‐acetyl glucosamine protein modification (O‐GlcNAcylation) is increased in host cells after Stx exposure and the subsequent endoplasmic reticulum (ER) stress response. The elevated O‐GlcNAcylation resulted in elevated inflammatory and apoptotic processes. Inhibition of O‐GlcNAcylation with OSMI‐1 protected cells from the Stx2a‐induced damage. In mice intoxicated with Stx2a, OSMI‐1 treatment reduced kidney damage and increased mouse survival.

## Graphical abstract

A. Melton‐Celsa and R. A. Bova discuss the role of O‐linked N‐acetyl glucosamine protein modification in Shiga toxin‐mediated pathogenesis as reported by SK Park and colleagues, in this issue of *EMBO Mol Med*.

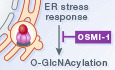

Shiga toxin (Stx)‐producing *E. coli* are food‐ and water‐borne causes of hemorrhagic colitis and hemolytic uremic syndrome (HUS). Production of Stx2a is particularly linked to HUS development (Melton‐Celsa, 2014; Scheutz, 2014). The Stxs consist of a catalytically active A subunit and a pentamer of B subunits that bind to target cell receptors (globotriaosylceramide, Gb3). Treatment for STEC infection is limited to supportive therapies because antibiotic regimens can increase the chance of developing HUS (Tarr *et al*, 2018). Potential interventions for STEC infection in development consist largely of antitoxin antibodies or vaccines, and receptor‐blocking analogs (Mühlen & Dersch, 2020). An anticomplement therapy that is successful in noninfectious HUS, Eculizumab, has not consistently demonstrated similar benefits for STEC‐related HUS (Mühlen & Dersch, 2020). However, Lee *et al* describe a novel strategy for interrupting Stx2a‐based damage and death in multiple cell types and organoids, and demonstrated protection of mice injected with Stx2a by an inhibitor of O‐linked N‐acetyl glucosamine protein modification O‐GlcNAcylation, OSMI‐1 (Lee *et al*, 2021). Protein modification by the addition of O‐GlcNAc affects many cellular processes, including the cell cycle and stress responses (Martinez *et al*, 2017; Estevez *et al*, 2020). The addition of O‐GlcNAc to proteins is facilitated by O*‐*GlcNAc transferase (OGT), which can be inhibited by OSMI‐1 (Alteen *et al*, 2021).

The decision to test whether OSMI‐1 could protect from Stx2a‐mediated damage came from the authors’ original finding that THP‐1 cells treated with Stx2a had increased levels of O*‐*GlcNAcylation associated with protein in cell lysates (Lee *et al*, 2021). This result revealed yet another cellular process altered by Stx intoxication. The Stxs, which travel through the cell in a retrograde manner from the endosome to the Golgi to the endoplasmic reticulum (ER), are known to induce both ER and ribotoxic stress responses in addition to halting protein synthesis in susceptible cells [see Fig 1 and (Lee *et al*, 2016)]. The increase in O‐GlcNAcylation observed by Lee *et al* may well be a reaction by the cell to ER stress induced by Stx2a; as such, O‐GlcNAcylation has been shown to decrease injury due to ER stress [see review (Martinez *et al*, 2017)]. Importantly, the authors showed that a catalytically inactive version of Stx2a that cannot target the ribosome was unable to increase O‐GlcNAcylation (Lee *et al*, 2021). This study demonstrated that in addition to an ER stress response, that an overall increase in O‐GlcNAc levels occurred, and that both pro‐inflammatory (p65) and pro‐apoptotic (Akt and Bad) signaling proteins were O‐GlcNAcylated, their phosphorylation status altered, and function increased. These pro‐inflammatory and apoptotic cellular changes could be inhibited by pretreatment of the THP‐1 cells with OSMI‐1 or an inhibitory RNA directed toward OGT.

**Figure 1:**
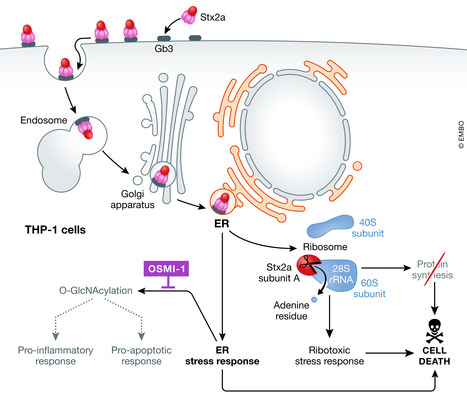
Simplified diagram of Stx2a translocation through the cell and the effect on cellular processes After binding to receptor Gb3, Stx2a traffics in a retrograde manner to the endoplasmic reticulum (ER) where it induces ER stress and O‐GlcNAcylation responses. The catalytically active A subunit of the toxin is released into the cytoplasm where it cleaves an adenine residue from the 28S rRNA, and, as a result, halts protein synthesis. OSMI‐1, an O‐GlcNAcylation transferase (OGT) inhibitor, prevents Stx2a‐mediated O‐GlcNAcylation and consequent pro‐inflammatory and apoptotic responses, thus protecting THP‐1 cells from Stx2a. [This is a simplified diagram of the effects of Stx2 on a cell, for a review see (Lee *et al*, 2016)].

The authors found a similar O‐GlcNAcylation response in endothelial cells, which are the target cell type in HUS. They used primary human renal proximal tubular endothelial cells (HRPTEpi) for these studies and demonstrated that the inflammatory cytokine and chemokine response to Stx2a were reduced by OSMI‐1 treatment, thereby showing that the response observed in THP‐1 cells was conserved in cells highly sensitive to Stx2a. The authors then moved to three‐dimensional (3D) human‐mini‐kidney spheroids and induced pluripotent stem cells (iPSC)‐derived renal organoids and demonstrated downregulation of pro‐inflammatory and apoptotic signals, as well as kidney injury marker Kim‐1 induced by Stx2a when these models were treated with OSMI‐1.

With a plethora of data supporting the hypothesis that inhibition of O‐GlcNAcylation is protective to cells and organoids *in vitro*, the authors used a mouse toxin injection model to test the potential protective efficacy of OSMI‐1 *in vivo*. Mice were injected daily with OSMI‐1 starting from the day before intoxication. Mice given OSMI‐1 were significantly protected from weight loss, elevated kidney injury markers, and death as compared to animals given the vehicle control.

The results from this paper demonstrate that inhibition of O*‐*GlcNAcylation could be a possible target for treatment to mitigate the effects Stx. It will be exciting to observe whether similar findings can be demonstrated in an STEC infection model. Although OGT mutations may be lethal (Estevez *et al*, 2020), and OSMI‐1 may have some toxicity (Alteen *et al*, 2021), the limited time course of an STEC infection likely provides an acceptable framework to test inhibitors of the cellular O‐GlcNAcylation process in additional *in vivo* models.
